# Prognostic value of serial alactic base excess measurements in patients with sepsis: a retrospective cohort study

**DOI:** 10.3389/fmed.2026.1755874

**Published:** 2026-03-13

**Authors:** Dursun Elmas, Muhammet Cemal Kizilarslanoglu

**Affiliations:** 1 Department of Internal Medicine, Division of Intensive Care, University of Health Sciences Türkiye, Konya City Hospital, Konya, Türkiye; 2 Department of Internal Medicine, Division of Geriatrics, University of Health Sciences Türkiye, Konya City Hospital, Konya, Türkiye

**Keywords:** alactic base excess, critical care, lactate, mortality, sepsis

## Abstract

**Background:**

This study evaluated the prognostic significance of serial alactic base excess (ABE) measurements in patients with sepsis.

**Methods:**

We conducted a retrospective cohort study including 521 adult patients with sepsis. Arterial blood gas analyses obtained at 0, 6, 12, 24, and 48 h were used to calculate ABE. Patients were classified into three trajectory groups: persistently low ABE (ABE < 0 at all time points), normalizing ABE (initial ABE < 0 with recovery to ≥0 by 48 h), and normal ABE (ABE ≥ 0 at admission). The primary outcome was 28-day mortality.

**Results:**

Overall 28-day mortality was 35.9%. Non-survivors exhibited more severe metabolic derangement at admission, with higher lactate levels (median 4.22 [2.89–5.31] vs. 3.66 [2.31–4.82] mmol/L) and more negative base excess values (median −6.00 [−8.54 to −3.88] vs. −5.09 [−7.63 to −2.71] mmol/L) (both *p* < 0.001). Admission ABE was significantly lower in non-survivors and remained consistently reduced throughout the first 48 h (all *p* < 0.001). Patients with a persistently low ABE trajectory experienced the highest mortality compared with those whose ABE normalized (52.9% vs. 19.8%, *p* < 0.001). In multivariable Cox regression adjusted for age, sex, baseline eGFR, SOFA score, and APACHE II score, persistently low ABE independently predicted 28-day mortality (adjusted HR 2.539; 95% CI 1.510–4.267; *p* < 0.001). Furthermore, each 1 mmol/L increase in ΔABE over 48 h was associated with a 25% relative reduction in mortality risk (adjusted HR 0.750; 95% CI 0.633–0.889; *p* = 0.001). At ICU admission, ABE showed numerically higher discrimination for 28-day mortality than BE (AUC 0.671 vs. 0.639), although this difference did not reach statistical significance (*p* = 0.057).

**Conclusion:**

Serial ABE trajectories provide independent prognostic information in sepsis. Failure to normalize ABE within the first 48 h identifies a high-risk phenotype associated with markedly increased short-term mortality.

## 1 Introduction

Sepsis is a life-threatening organ dysfunction caused by a dysregulated host response to infection and remains a leading cause of mortality in intensive care units worldwide (1, 2). Early identification of high-risk patients is essential to guide timely resuscitation and organ support strategies (3). Serum lactate is widely used as a marker of tissue hypoperfusion and is incorporated into contemporary sepsis guidelines (4). Although elevated lactate levels are consistently associated with poor outcomes (5), lactate is not a specific indicator of cellular hypoxia and may be influenced by *β*-adrenergic stimulation, impaired hepatic clearance, or altered metabolism during systemic inflammation (3–6). Accordingly, clinical trials focusing solely on lactate-guided resuscitation have demonstrated variable survival benefits, highlighting that lactate normalization does not necessarily reflect comprehensive metabolic recovery (4–6).

Metabolic acidosis in sepsis arises from both lactate accumulation and the retention of non-lactate (“fixed”) acids, commonly driven by acute kidney injury, unmeasured anions, and chloride-rich fluid administration (3). Standard base excess (BE) reflects the overall metabolic component of acid–base disturbance but does not differentiate between these mechanisms. To address this limitation, alactic base excess (ABE), calculated as BE plus lactate (ABE = BE + lactate), has been proposed as a bedside parameter that isolates the fixed-acid component of metabolic derangement (3–7). Physiologically, ABE reflects the kidney’s capacity to compensate for acid load and regulate strong ion differences, thereby providing insight into renal metabolic reserve and systemic acid handling (7, 8). Persistently negative ABE values suggest ongoing accumulation of unmeasured acids or impaired renal tubular function, whereas markedly positive values may indicate metabolic alkalosis or iatrogenic overcorrection (9, 10). Recent observational data suggest that deviations of ABE from the neutral range are associated with adverse outcomes, underscoring its potential prognostic relevance (1–3).

Several studies have reported associations between admission ABE and mortality (11, 12) or renal replacement therapy (RRT) requirements in critically ill populations (13, 14). However, these investigations have largely relied on single time-point measurements obtained at presentation. Sepsis is a dynamic syndrome characterized by evolving organ dysfunction and metabolic adaptation, and static baseline values may inadequately capture the trajectory of recovery or deterioration. While serial lactate clearance is routinely used to monitor resuscitation response (2), far less attention has been paid to the temporal behavior of fixed-acid metabolism. Whether changes in ABE over time provide incremental prognostic information beyond lactate remains largely unexplored.

We hypothesized that failure to correct ABE during the early phase of intensive care reflects a distinct phenotype of metabolic and renal dysfunction associated with poor outcomes, independent of lactate normalization. Accordingly, this study aimed to evaluate the prognostic value of serial ABE measurements in septic intensive care unit (ICU) patients by classifying individuals according to dynamic ABE trajectories (persistent versus normalizing) and examining their association with 28-day mortality.

## 2 Methods

### 2.1 Ethics approval and consent to participate

This study was conducted in accordance with the principles of the Declaration of Helsinki and was approved by the Clinical Research Ethics Committee of Karatay University (Approval No: 2025/044; Document Date and Number: 14.04.2025–107719). The requirement for informed consent was waived by the ethics committee because the study used existing anonymized data from the hospital automation system, with no identifying information accessed. Data were accessed for research purposes on between January 1, 2019 and December 31, 2024.

### 2.2 Study design, objective, and patients

This single-center retrospective cohort study aimed to investigate the prognostic value of serial ABE measurements during the first 48 h of ICU admission in patients with sepsis. We specifically examine whether persistent alactic metabolic acidosis (negative ABE) vs. normalization of ABE correlates with 28-day mortality and secondary endpoints (organ support requirements).

We also compare the predictive performance of ABE trends against conventional lactate metrics. We aimed to test the hypothesis that failure to correct ABE (i.e., sustained negative ABE) is associated with higher mortality, independent of lactate levels and other confounders.

**Figure 1:**
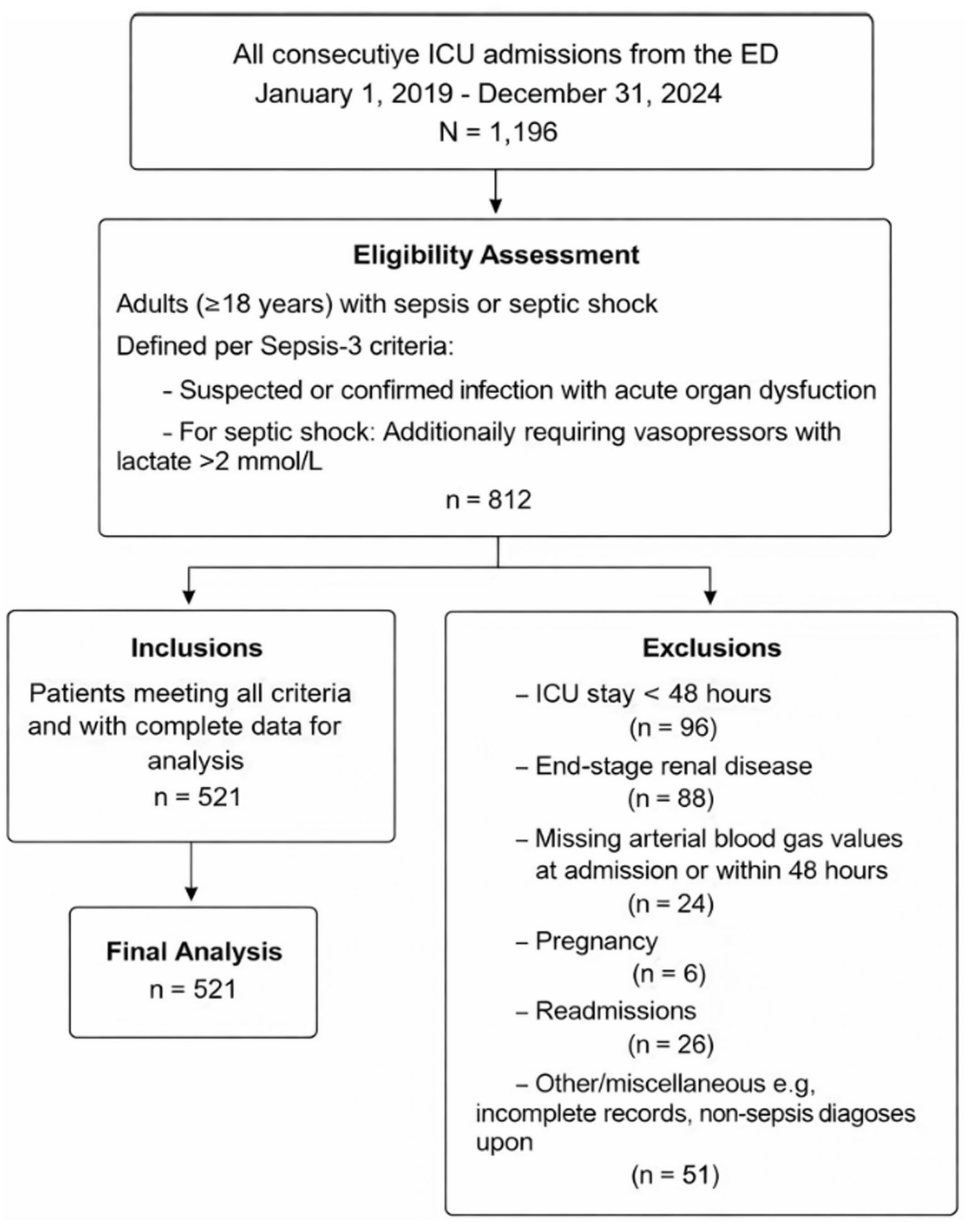
Patient flow diagram for the retrospective cohort study. Patient flow diagram detailing the selection process for the retrospective cohort study of serial alactic base excess (ABE) in septic patients.

We screened all ICU admissions from the emergency department between January 1, 2019 and December 31, 2024 for inclusion in this study. Patients were eligible if they were adults (≥18 years) admitted with a diagnosis of sepsis or septic shock as defined by the Sepsis-3 consensus (suspected or confirmed infection with acute organ dysfunction for sepsis, and additionally requiring vasopressors with lactate >2 mmol/L for septic shock) (15). We excluded patients who had an ICU stay of <48 h (insufficient time for serial laboratory assessment), end-stage renal disease on chronic dialysis or prior renal transplantation (as ABE dynamics would be substantially altered), pregnancy, or ICU readmissions (only the first ICU admission was considered). In addition, patients were excluded if arterial blood gas measurements were unavailable at ICU admission (0 h) or if no follow-up arterial blood gas measurement was available within the first 48 h, as at least two measurements were required to assess early ABE dynamics. Patient selection is illustrated in the flow diagram (Figure 1).

### 2.3 Data collection

Using electronic health records, we extracted demographic data (age and sex) and comorbidities (diabetes, hypertension, COPD, CKD, and malignancy). The severity of illness was assessed using the Sequential Organ Failure Assessment (SOFA) and APACHE II scores upon admission (16). We recorded initial vital signs (blood pressure, heart rate, etc.) and laboratory values, including complete blood count, electrolytes, creatinine, estimated GFR, bilirubin, albumin, and C-reactive protein (CRP). Arterial blood gas (ABG) measurements at 0, 6, 12, 24, and 48 h after ICU admission were collected. Each ABG provided arterial pH, partial pressures (PaO₂, PaCO₂), bicarbonate, standard base excess (BE in mmol/L), and lactate (mmol/L). From these, we calculated ABE = BE + lactate at each time point (7). For example, if BE = −5 mmol/L and lactate = 4 mmol/L, ABE = −1 mmol/L. ABE thus represents the “fixed” (non-lactic) component of metabolic deviation. We also noted interventions, including the use of mechanical ventilation, vasopressors (type and duration), and initiation of RRT during the ICU stay. The requirement for invasive mechanical ventilation was defined as endotracheal intubation and ventilatory support initiated at any time during the index ICU stay, regardless of timing relative to ICU admission. AKI was identified and staged via KDIGO criteria based on serum creatinine or urine output (17). AKI was defined and staged according to the KDIGO criteria using both serum creatinine and urine output when available. Because urine output in critically ill patients may be influenced by diuretic administration, serum creatinine–based criteria were prioritized for AKI staging in cases of potential ambiguity. Diuretic exposure was not uniformly available in this retrospective dataset and was therefore not included as a separate adjustment variable. The need for RRT was determined by treating physicians (e.g., severe AKI with oliguria or refractory acidosis). All laboratory and arterial blood gas analyses were performed in the hospital’s core laboratories; lactate measurements were conducted on heparinized arterial blood samples using the ABL90 FLEX PLUS blood gas analyzer (Radiometer Medical ApS, Brønshøj, Denmark).

### 2.4 ABE trend groups

We classified patients into three mutually exclusive categories based on their ABE values from 0 to 48 h: (1) Persistently low ABE: ABE remained negative (<0) at all measured time points up to 48 h; (2) Normalizing ABE: ABE was negative at admission (0 h), but improved to ≥0 by 48 h; (3) Normal ABE: ABE was already ≥zero at admission (indicating no significant fixed acidosis initially). These groups were designed to reflect persistent versus resolving metabolic acidosis due to non-lactate causes. The 0 mmol/L threshold was selected *a priori* as it physiologically denotes the absence of fixed metabolic acidosis (aligning with normal BE ranges) and statistically maximized separation between trajectory groups in exploratory analysis (e.g., mortality differences *p* < 0.001) (12–23). This cutoff prioritizes detection of normalization over high-specificity single-point prediction (e.g., −3.63 mmol/L for mortality (12)), enabling assessment of metabolic recovery dynamics. Additionally, we computed the change in ABE from 0 to 48 h (ΔABE = ABE 48 h – ABE 0 h) for each patient as a continuous variable (positive ΔABE indicating improvement toward alkalinity or less acidosis). Patients were also stratified by lactate clearance for comparison; we calculated 6-h and 24-h lactate clearance (%) as follows: [(initial lactate – subsequent lactate)/initial lactate] × 100%.

Trajectory classification was based on arterial blood gas measurements obtained at predefined nominal time points (0, 6, 12, 24, and 48 h after ICU admission). When multiple arterial blood gas samples were available within a given time window, the measurement closest to the nominal time point was selected for analysis. Thus, all available blood gas data within the first 48 h were incorporated through this structured time-point approach rather than relying solely on baseline and 48-h values. Importantly, patients who demonstrated ABE normalization (≥0 mmol/L) at any intermediate time point were not classified as having a persistently low ABE trajectory, even if subsequent measurements showed renewed negativity, in order to avoid misclassification driven by transient metabolic fluctuations.

### 2.5 Outcomes

The primary outcome was 28-day all-cause mortality. We also evaluated ICU mortality (during the index ICU stay) for comparison. Secondary outcomes included: requirement of RRT during ICU (yes/no), need for invasive mechanical ventilation, total duration of vasopressor support (days), and ICU length of stay. We recorded development of AKI (new AKI or progression to a higher KDIGO stage) as a related outcome, since ABE may be linked to renal function.

### 2.6 Statistical analysis

All statistical analyses were performed using IBM SPSS Statistics for Windows, version 24.0 (IBM Corp., Armonk, NY, USA). Continuous variables were assessed for normality and are presented as median [interquartile range, IQR] or mean ± standard deviation, as appropriate. Categorical variables are expressed as counts and percentages. Between-group comparisons were performed using Student’s *t* test or Mann–Whitney *U* test for continuous variables and Chi-square or Fisher’s exact test for categorical variables, as appropriate.

For longitudinal assessment of lactate, BE, and ABE across repeated time points (0, 6, 12, 24, and 48 h), linear mixed-effects models with random intercepts for individual patients were applied to account for within-subject correlation. Time, mortality group (survivor vs. non-survivor), and their interaction (time × group) were included as fixed effects. This approach allowed evaluation of overall group differences and temporal trajectories while appropriately handling missing observations. Patients were not excluded due to missing intermediate blood gas measurements, provided that the minimum inclusion requirement was met; missing repeated measurements were handled using maximum likelihood estimation under a missing-at-random assumption, without imputation. Post-hoc pairwise comparisons were adjusted using Bonferroni correction to control for multiple testing. Descriptive comparisons at individual time points are presented in tables; however, inferential interpretation of repeated measures was based on mixed-effects modeling.

Cox proportional hazards regression models were used to examine the association between ABE dynamics and mortality. Primary predictors included ΔABE (modeled as a continuous variable per 1 mmol/L increase from admission to 48 h) and ABE trajectory groups (persistently low, normalizing, and normal ABE), with the normalizing group selected as the reference category. Initially, unadjusted (crude) models were constructed. Multivariable models were then adjusted for clinically relevant covariates known to influence sepsis outcomes, including age, sex, baseline eGFR, SOFA score, and APACHE II score. Proportional hazards assumptions were assessed using Schoenfeld residuals. Covariates were selected *a priori* based on established clinical relevance and prognostic importance in sepsis, in order to account for baseline demographics, illness severity, and renal function while avoiding overadjustment for potential mediators. Collinearity between severity scores was assessed, and model stability was confirmed by sensitivity analyses including SOFA and APACHE II separately.

Given the retrospective design, no a priori sample size calculation was performed; all eligible patients during the predefined study period were included. As supportive information, an a posteriori power analysis was conducted using G*Power (version 3.1.9.7). Based on the observed standardized effect size for ΔABE between 28-day survivors and non-survivors (Cohen’s *d* = 0.243), the available group sizes (*n* = 360 and *n* = 161), and a two-sided *α* of 0.05, the achieved statistical power was 89.95%, indicating adequate precision for the primary outcome. In addition, the number of events satisfied recommended events-per-variable criteria for multivariable Cox modeling.

Results are reported as hazard ratios (HR) with 95% confidence intervals (CI). All statistical tests were two-sided, and a *p* value < 0.05 was considered statistically significant.

## 3 Results

### 3.1 Patient characteristics

A total of 521 patients met the inclusion criteria (median age 61 [49–72] years; 50.9% female). Overall disease severity was high, with a median SOFA score of 9 [6–13] and APACHE II score of 18 [14–24]. There were no significant differences in age or sex between survivors and non-survivors.

Non-survivors exhibited a substantially higher burden of organ dysfunction. Although admission creatinine levels were slightly lower in non-survivors (median 1.33 [0.90–1.66] vs. 1.51 [0.90–1.67] mg/dL, *p* = 0.023), they developed acute kidney injury more frequently during ICU follow-up (43.5% vs. 16.4%, *p* < 0.001) and required renal replacement therapy more often (23.0% vs. 8.6%, *p* < 0.001). Mechanical ventilation was required in nearly all non-survivors (100% vs. 7.2% among survivors, *p* < 0.001). Illness severity scores were also significantly higher in non-survivors (median SOFA 14 [10–17] vs. 8 [5–11]; APACHE II 22 [18–27] vs. 17 [13–22]; both *p* < 0.001). Baseline clinical and laboratory characteristics stratified by ICU mortality are summarized in Table 1, and comparisons according to 28-day mortality are presented in Table 2. All continuous variables are reported as median [interquartile range].

**Table 1:** Comparison of clinical and laboratory parameters of the patients according to the ICU mortality status.

Parameters	All patients (*n* = 521)	Discharged (*n* = 360)	Died (*n* = 161)	*p*-value
Female sex, *n* (%)	265 (50.9)	182 (50.6)	83 (51.6)	0.833
Age, years	61 [49–72]	60 [48–71]	63 [52–74]	0.969
Comorbidities, *n* (%)				
Diabetes mellitus	247 (47.4)	166 (46.1)	81 (50.3)	0.375
Hypertension	247 (47.4)	165 (45.8)	82 (50.9)	0.282
COPD	271 (52.0)	194 (53.9)	77 (47.8)	0.201
Chronic kidney disease	132 (25.3)	93 (25.8)	39 (24.2)	0.696
Malignancy	272 (52.2)	182 (50.6)	90 (55.9)	0.259
Renal replacement therapy	68 (13.1)	31 (8.6)	37 (23.0)	<0.001
Acute kidney injury	129 (24.8)	59 (16.4)	70 (43.5)	<0.001
Mechanical ventilation support, *n* (%)	187 (35.9)	26 (7.2)	161 (100)	<0.001
SOFA score	9 [6–13]	8 [5–11]	14 [10–17]	<0.001
APACHE II score	18 [14–24]	17 [13–22]	22 [18–27]	<0.001
ICU length of stay, days	14 [9–21]	14 [8–20]	16 [11–23]	0.001
Laboratory findings				
eGFR (mL/min/1.73 m^2^)	67.9 [45.0–89.0]	68.8 [48.0–92.0]	64.7 [41.0–83.0]	0.010
pH	7.29 [7.23–7.35]	7.31 [7.25–7.36]	7.24 [7.18–7.30]	<0.001
CRP (mg/L)	106 [62–168]	95 [55–142]	130 [81–189]	<0.001
Creatinine (mg/dL)	1.47 [1.10–1.71]	1.51 [0.90–1.67]	1.33 [0.90–1.66]	0.023
Lactate 0 h	3.96 [2.41–5.02]	3.76 [2.29–4.71]	4.22 [2.89–5.31]	<0.001
BE 0 h	−5.47 [−8.12 to −3.04]	−5.20 [−7.63 to −2.71]	−6.01 [−8.54 to −3.88]	<0.001
ABE 0 h	−1.12 [−3.42 to 1.81]	−0.86 [−3.01 to 2.09]	−1.58 [−3.91 to 0.84]	<0.001
Lactate 6 h	3.54 [2.12–4.83]	3.37 [2.01–4.62]	3.85 [2.51–5.02]	<0.001
BE 6 h	−4.76 [−7.41 to −1.92]	−4.45 [−6.98 to −1.61]	−5.33 [−8.03 to −2.28]	<0.001
ABE 6 h	−0.90 [−3.18 to 2.44]	−0.60 [−2.71 to 2.81]	−1.21 [−3.69 to 1.84]	<0.001
Lactate 12 h	2.99 [1.92–4.21]	2.82 [1.81–3.97]	3.24 [2.11–4.48]	<0.001
BE 12 h	−4.14 [−6.32 to −1.04]	−3.73 [−5.98 to −0.72]	−5.02 [−7.14 to −2.01]	<0.001
ABE 12 h	−0.88 [−2.74 to 2.59]	−0.50 [−2.41 to 2.87]	−1.54 [−3.12 to 1.33]	<0.001
Lactate 24 h	2.05 [1.31–3.02]	1.95 [1.21–2.74]	2.52 [1.61–3.44]	<0.001
BE 24 h	−2.77 [−5.41 to 0.52]	−2.50 [−4.93 to 0.71]	−4.09 [−6.12 to −1.01]	<0.001
ABE 24 h	−0.78 [−2.83 to 2.41]	−0.53 [−2.41 to 2.76]	−1.26 [−3.11 to 1.62]	<0.001
Lactate 48 h	1.28 [0.92–2.11]	1.23 [0.88–1.94]	1.48 [1.01–2.36]	0.001
BE 48 h	0.14 [−2.91 to 2.36]	0.44 [−2.41 to 2.68]	−2.81 [−4.75 to 1.02]	<0.001
ABE 48 h	0.50 [−2.63 to 2.91]	0.88 [−2.21 to 3.14]	−0.62 [−3.19 to 1.68]	<0.001
ΔABE	0.82 [−1.21 to 3.44]	0.95 [−0.98 to 3.91]	0.51 [−1.84 to 2.66]	0.006
ABE trajectory, *n* (%)				
Normalizing	159.0 (30.5)	116.0 (32.2)	43.0 (26.7)	<0.001
Normal	174.0 (33.4)	140.0 (38.9)	34.0 (21.1)	
Persistent	188.0 (36.1)	104.0 (28.9)	84.0 (52.2)	
Delta ABE, *n* (%)				
Increased	389.0 (74.7)	281.0 (78.1)	108.0 (67.1)	0.017
Decreased	129.0 (24.8)	78.0 (21.7)	51.0 (31.7)	
Stable	3.0 (0.6)	1.0 (0.3)	2.0 (1.2)	

Values are presented as median [interquartile range] or *n* (%), unless otherwise indicated. Lactate, base excess (BE), and alactic base excess (ABE) are expressed in mmol/L. Between-group comparisons at individual time points are shown descriptively using Mann–Whitney *U* test for continuous variables and Chi-square or Fisher’s exact test for categorical variables, as appropriate. Longitudinal comparisons across repeated measurements were performed using linear mixed-effects models, as described in the Statistical Analysis section. Statistically significant *p*-values (<0.05). DM, diabetes mellitus; HT, hypertension; COPD, chronic obstructive pulmonary disease; CKD, chronic kidney disease; RRT, renal replacement therapy; AKI, acute kidney injury; CRP, C-reactive protein; SOFA, Sequential Organ Failure Assessment; APACHE II, Acute Physiology and Chronic Health Evaluation II; BE, base excess; ABE, alactic base excess; ΔABE, change in ABE from admission to 48 h.

**Table 2:** Comparison of clinical and laboratory parameters of the patients according to the 28-day mortality status.

Parameters	Alive (*n* = 334)	Died (*n* = 187)	*p*-value
Female sex, *n* (%)	164 (49.1)	101 (54.0)	0.282
Age, years	59.5 [47–71]	64 [52–75]	0.382
Comorbidities, *n* (%)			
Diabetes mellitus	154 (62.3)	93 (49.7)	0.427
Hypertension	159 (47.6)	88 (47.1)	0.905
COPD	176 (52.7)	95 (50.8)	0.678
Chronic kidney disease	90 (26.9)	42 (22.5)	0.259
Malignancy	168 (50.3)	104 (55.6)	0.244
Renal replacement therapy	28 (8.4)	40 (21.4)	<0.001
Acute kidney injury	54 (16.2)	75 (40.1)	<0.001
Mechanical ventilation support, *n* (%)	20 (6.0)	167 (89.3)	<0.001
SOFA score	8 [5–11]	14 [10–17]	<0.001
APACHE II score	17 [13–22]	22 [18–27]	<0.001
ICU length of stay, days	14 [8–20]	16 [11–23]	0.001
Laboratory findings			
eGFR (mL/min/1.73 m^2^)	68.6 [47.0–91.0]	66.1 [42.0–84.0]	0.038
pH	7.31 [7.25–7.36]	7.25 [7.19–7.31]	<0.001
CRP (mg/L)	94.6 [56.0–141.0]	124.9 [78.0–186.0]	<0.001
Lactate 0 h	3.66 [2.31–4.82]	4.22 [2.89–5.31]	<0.001
BE 0 h	−5.09 [−7.63 to −2.71]	−6.00 [−8.54 to −3.88]	<0.001
ABE 0 h	−0.77 [−3.01 to 2.09]	−1.62 [−3.91 to 0.84]	<0.001
Lactate 6 h	3.29 [2.01–4.62]	3.84 [2.51–5.02]	<0.001
BE 6 h	−4.34 [−6.98 to −1.61]	−5.35 [−8.03 to −2.28]	<0.001
ABE 6 h	−0.51 [−2.71 to 2.81]	−1.23 [−3.69 to 1.84]	<0.001
Lactate 12 h	2.75 [1.81–3.97]	3.24 [2.11–4.48]	<0.001
BE 12 h	−3.66 [−5.98 to −0.72]	−5.02 [−7.14 to −2.01]	<0.001
ABE 12 h	−0.39 [−2.41 to 2.87]	−1.50 [−3.12 to 1.33]	<0.001
Lactate 24 h	1.93 [1.21–2.74]	2.53 [1.61–3.44]	<0.001
BE 24 h	−2.38 [−4.93 to 0.71]	−4.20 [−6.12 to −1.01]	<0.001
ABE 24 h	−0.33 [−2.41 to 2.76]	−1.36 [−3.11 to 1.62]	<0.001
Lactate 48 h	1.23 [0.88–1.94]	1.46 [1.01–2.36]	0.001
BE 48 h	0.55 [−2.41 to 2.68]	−3.15 [−4.75 to 1.02]	<0.001
ABE 48 h	1.09 [−2.21 to 3.14]	−0.69 [−3.19 to 1.68]	<0.001
ΔABE	1.00 [−0.98 to 3.91]	0.52 [−1.84 to 2.66]	0.001
ABE trajectory, *n* (%)			
Normalizing	108 (32.3)	51 (27.3)	<0.001
Normal	137 (41.0)	37 (19.8)	
Persistent	89 (26.6)	99 (52.9)	
Delta ABE, *n* (%)			
Increased	264 (79.0)	125 (66.8)	0.007
Decreased	69 (20.7)	60 (32.1)	
Stable	1 (0.3)	2 (1.1)	

Values are presented as median [interquartile range] or *n* (%), unless otherwise indicated. Lactate, base excess (BE), and alactic base excess (ABE) are expressed in mmol/L. Between-group comparisons at individual time points are shown descriptively using Mann–Whitney *U* test for continuous variables and Chi-square or Fisher’s exact test for categorical variables, as appropriate. Inferential analyses of longitudinal trends and group–time interactions were performed using linear mixed-effects models, which account for repeated measurements and multiple comparisons, as detailed in the Statistical Analysis section. Statistically significant *p*-values (<0.05) are indicated in bold. DM, diabetes mellitus; HT, hypertension; COPD, chronic obstructive pulmonary disease; CKD, chronic kidney disease; RRT, renal replacement therapy; AKI, acute kidney injury; CRP, C-reactive protein; SOFA, Sequential Organ Failure Assessment; APACHE II, Acute Physiology and Chronic Health Evaluation II; eGFR, estimated glomerular filtration rate; BE, base excess; ABE, alactic base excess; ΔABE, change in ABE from admission to 48 h. Bold values indicate statistically significant results (*p* < 0.05).

### 3.2 Serial arterial blood gas profiles

At ICU admission, non-survivors demonstrated more severe metabolic derangement. Arterial pH was lower (median 7.25 [7.19–7.31] vs. 7.31 [7.25–7.36], *p* < 0.001), BE was more negative (−6.00 [−8.54 to −3.88] vs. −5.09 [−7.63 to −2.71] mmol/L, *p* < 0.001), and lactate levels were modestly higher (4.22 [2.89–5.31] vs. 3.66 [2.31–4.82] mmol/L, *p* < 0.001). Initial ABE was also significantly lower in patients who subsequently died (median −1.62 [−3.91 to 0.84] vs. −0.77 [−3.01 to 2.09] mmol/L, *p* < 0.001) (Table 3).

**Table 3:** Crude and adjusted regression models showing the relationships between ΔABE, ABE level trends, and mortality status.

Parameter	ICU mortality - unadjusted HR (95% CI)	*p*-value	ICU mortality - adjusted HR (95% CI)	*p*-value	28-day mortality - unadjusted HR (95% CI)	*p*-value	28-Day mortality - adjusted HR (95% CI)	*p*-value
ΔABE (per mmol/L increase)	0.838 (0.732–0.959)	0.010	0.779 (0.648–0.937)	0.008	0.805 (0.705–0.919)	0.001	0.750 (0.633–0.889)	0.001
Persistent low ABE trend*	3.326 (2.074–5.334)	<0.001	1.856 (1.077–3.198)	0.026	4.119 (2.594–6.540)	<0.001	2.539 (1.510–4.267)	<0.001

Unadjusted models represent crude associations between each predictor and mortality. Adjusted models were constructed a priori to account for baseline demographics, illness severity, and renal function, including age, sex, estimated glomerular filtration rate (eGFR), Sequential Organ Failure Assessment (SOFA), and Acute Physiology and Chronic Health Evaluation II (APACHE II) scores. Proportional hazards assumptions were verified using Schoenfeld residuals. Sensitivity analyses excluding SOFA to address potential collinearity with APACHE II yielded similar results (ICU mortality adjusted HR 1.856 [95% CI 1.077–3.198], *p* = 0.026; 28-day mortality adjusted HR 2.539 [95% CI 1.510–4.267], *p* < 0.001), confirming model stability. The normalizing ABE group was used as the reference category for ABE trajectory comparisons. *p*-values < 0.05 were considered statistically significant. HR, hazard ratio; CI, confidence interval; ΔABE, change in alactic base excess between admission and 48 h; ABE, alactic base excess; ICU, intensive care unit; eGFR, estimated glomerular filtration rate; SOFA, Sequential Organ Failure Assessment; APACHE II, Acute Physiology and Chronic Health Evaluation II. *Compared with the normalizing ABE group (reference category).

Over the first 48 h, lactate concentrations declined in both groups but remained consistently higher in non-survivors at all measured time points (0, 6, 12, 24, and 48 h; all *p* < 0.001). In contrast, BE improved substantially in survivors, increasing from −5.09 [−7.63 to −2.71] mmol/L at admission to 0.55 [−2.41 to 2.68] mmol/L at 48 h, whereas BE in non-survivors remained persistently negative (from −6.00 [−8.54 to −3.88] to −3.15 [−4.75 to 1.02] mmol/L). ABE trajectories showed the most pronounced separation between groups: survivors exhibited progressive normalization of ABE, reaching a median of 1.09 [−2.21 to 3.14] mmol/L at 48 h, while non-survivors remained below zero through 24 h and were still slightly negative at 48 h (median −0.69 [−3.19 to 1.68] mmol/L). Differences in lactate, BE, and ABE between survivors and non-survivors were statistically significant at all time points (Table 2).

**Figure 2:**
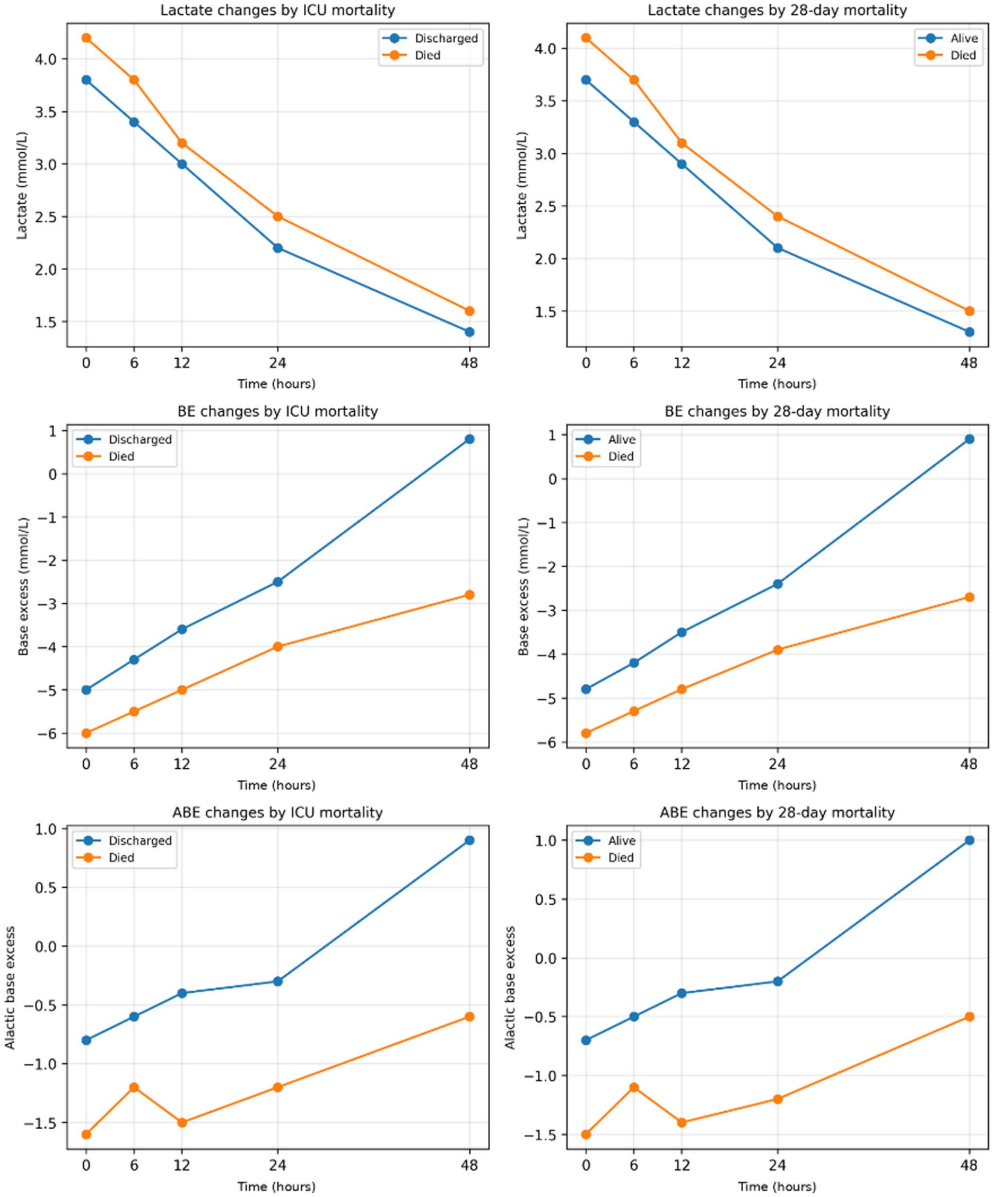
This figure shows the changes in lactate, BE, and ABE levels within the first 48 h in the ICU according to mortality status. Changes in lactate, base excess (BE), and alactic base excess (ABE) levels during the first 48 h in the ICU according to survival status. Lines represent median values at each time point. Individual variability (min–max ranges) is reported in Supplementary Table 2. All values (lactate, BE, and ABE) are expressed in mmol/L. While lactate decreased in all patients, only survivors achieved normalization of BE and ABE. Persistently negative ABE was strongly associated with both ICU and 28-day mortality.

Figure 2 illustrates the serial changes in lactate, BE, and ABE over 48 h. Lactate declined in both groups but remained higher in non-survivors. Conversely, BE and ABE improved markedly in survivors, whereas both parameters remained negative in non-survivors (group and time × group interaction effects, all *p* < 0.001).

### 3.3 Outcomes according to ABE trajectory

Patients were classified into three groups based on ABE dynamics: persistent low ABE (*n* = 188, 36.1%), normalizing ABE (*n* = 159, 30.5%), and normal ABE (*n* = 174, 33.4%). Twenty-eight-day mortality differed significantly among groups (52.9, 19.8, and 26.6% for persistent low, normalizing, and normal ABE, respectively; *p* < 0.001). Thus, patients with persistently low ABE had approximately double the mortality risk compared with those whose ABE normalized within 48 h.

The persistently low ABE group exhibited a markedly higher burden of organ dysfunction, including more frequent acute kidney injury (43.5% vs. 16.4% in the normalizing group, *p* < 0.001) and greater need for renal replacement therapy (23.0% vs. 8.6%, *p* < 0.001). These patients also had higher illness severity scores and longer ICU stays (median 16 [11–23] vs. 14 [8–20] days, *p* = 0.001). At admission, they presented with higher lactate levels, more negative BE, and consequently a substantially lower ABE compared with the other groups. In contrast, patients in the normalizing ABE group showed progressive improvement in both BE and lactate over 24–48 h. Patients with initially normal ABE generally had lower severity of shock but still experienced non-negligible mortality, indicating that absence of early fixed-acid acidosis does not preclude adverse outcomes when other organ failures are present.

Sensitivity analyses using alternative ABE thresholds (−1 and −2 mmol/L) yielded consistent results, confirming the robustness of the primary classification (Supplementary Table 3).

### 3.4 Primary outcome: 28-day mortality

In univariable Cox regression, greater improvement in ABE over the first 48 h (ΔABE) was associated with lower 28-day mortality (HR 0.805 per mmol/L increase, 95% CI 0.705–0.919; *p* = 0.001). After adjustment for age, sex, baseline eGFR, SOFA score, and APACHE II score, ΔABE remained an independent predictor of survival (adjusted HR 0.750 per mmol/L increase, 95% CI 0.633–0.889; *p* = 0.001), indicating an approximately 25% relative reduction in mortality risk for each 1 mmol/L increase in ABE.

When analyzed categorically, a persistently low ABE trajectory was strongly associated with increased 28-day mortality compared with the normalizing group (unadjusted HR 4.119, 95% CI 2.594–6.540; *p* < 0.001). This association remained significant after multivariable adjustment (adjusted HR 2.539, 95% CI 1.510–4.267; *p* < 0.001). In contrast, patients with normal ABE at admission had mortality risks comparable to those in the normalizing group after adjustment (adjusted HR 1.07, 95% CI 0.66–1.75; *p* = 0.78).

Similar associations were observed for ICU mortality: persistently low ABE remained independently associated with death after adjustment (adjusted HR 1.856, 95% CI 1.077–3.198; *p* = 0.026). Notably, ABE dynamics demonstrated stronger prognostic discrimination than admission lactate alone; while each mmol/L increase in lactate was associated with a modest mortality risk increase (adjusted HR ~ 1.09), persistent ABE negativity conferred a substantially higher adjusted hazard; each mmol/L increase in lactate was associated with only a modest increase in mortality risk (adjusted HR approximately 1.09), whereas persistent ABE negativity conferred a substantially higher adjusted hazard.

**Figure 3:**
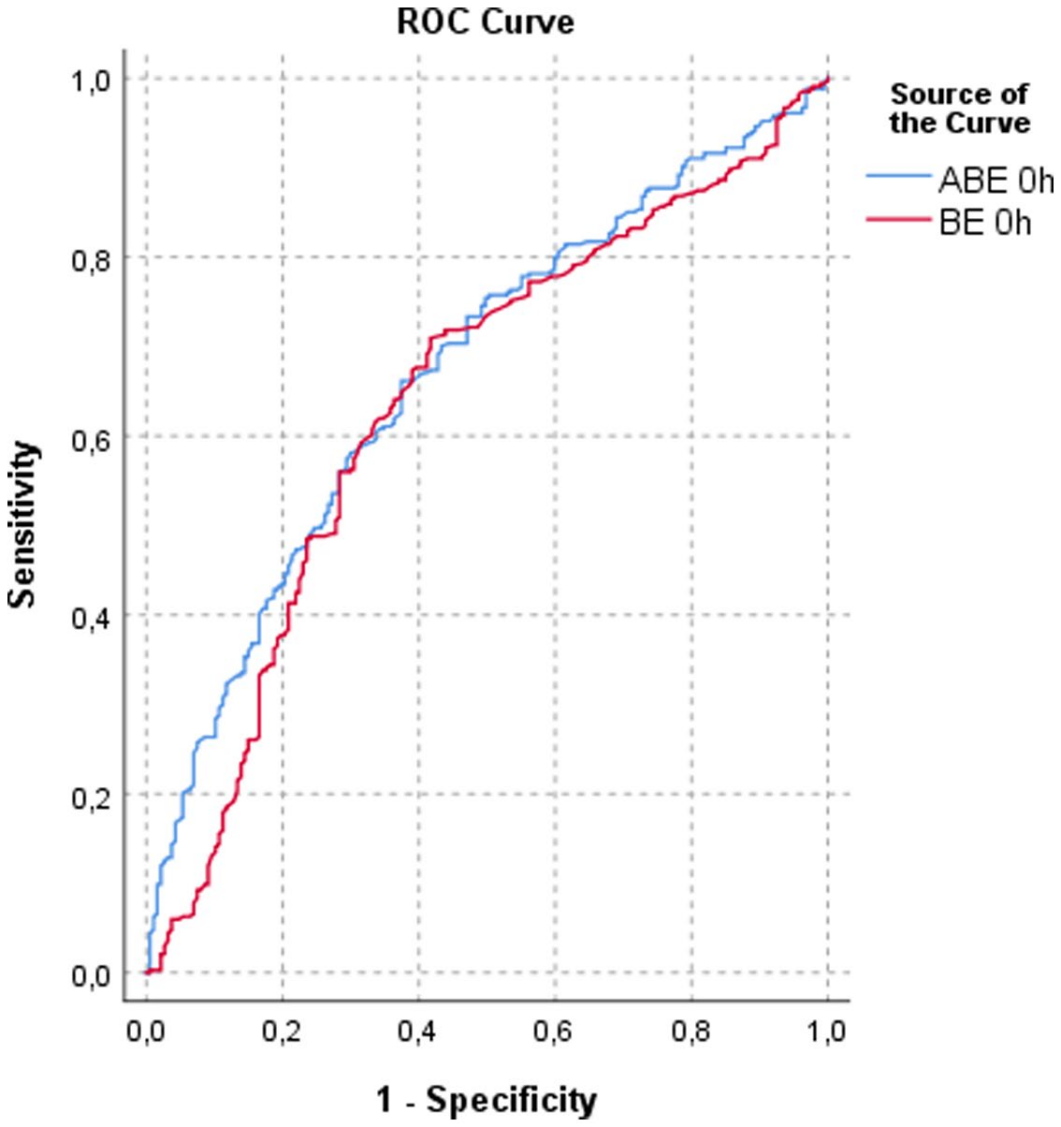
ROC curves comparing admission ABE 0 h and BE 0 h for prediction of 28-day mortality. ABE demonstrated modestly higher discrimination than BE (AUC 0.671 [95% CI 0.624–0.718] vs. 0.639 [95% CI 0.589–0.689]); however, the difference between curves was not statistically significant (ΔAUC = 0.032; *Z* = −1.907; *p* = 0.057).

Receiver operating characteristic analysis at ICU admission showed that ABE had numerically higher discriminatory ability for 28-day mortality compared with BE (AUC 0.671 vs. 0.639). However, pairwise comparison of ROC curves revealed that this difference did not reach statistical significance (ΔAUC = 0.032; *Z* = −1.907; *p* = 0.057) (Figure 3).

### 3.5 Secondary outcomes

Persistent alactic acidosis was associated with worse organ support requirements. Patients with persistently low ABE required renal replacement therapy more frequently and experienced higher rates of acute kidney injury during ICU follow-up. They also had longer ICU stays and greater dependence on invasive mechanical ventilation. Very low admission ABE values (e.g., <−5 mmol/L) were strongly associated with subsequent RRT requirement, further supporting the link between unresolved fixed-acid burden and impaired renal metabolic compensation, supporting the link between unresolved fixed-acid burden and impaired renal metabolic compensation.

## 4 Discussion

In this retrospective cohort of critically ill septic patients, we demonstrated that dynamic ABE trajectories provide robust prognostic information beyond conventional acid–base markers. The principal finding is that failure to normalize ABE within the first 48 h identifies a distinct high-risk phenotype associated with markedly increased 28-day mortality, independent of illness severity and baseline renal function. Moreover, incremental improvement in ABE over time was independently associated with survival, underscoring that metabolic recovery—rather than isolated correction of lactate—plays a critical role in patient outcomes.

A key observation of this study is the dissociation between lactate clearance and resolution of fixed metabolic acidosis. While lactate concentrations declined in most patients during early resuscitation, a substantial proportion of non-survivors remained in persistent base deficit as reflected by negative ABE. Importantly, this pattern should not be interpreted as a direct mechanistic link between lactate-related pathways and persistent ABE. Lactatemia encompasses a broad spectrum of processes, including type A and type B hyperlactatemia driven by adrenergic stimulation, aerobic glycolysis, microcirculatory alterations, and impaired clearance. In contrast, ABE primarily reflects the burden of non-lactate acids and unmeasured anions, often related to impaired renal acid handling and sustained tissue hypoperfusion. Consequently, lactate normalization may occur despite ongoing accumulation of fixed acids, underscoring that resolution of hyperlactatemia alone does not necessarily indicate global metabolic recovery (7–18). In contrast, ABE isolates the non-lactate component of metabolic acidosis and reflects ongoing accumulation of fixed acids and impaired renal or microcirculatory compensation (7–10). Our findings indicate that this unresolved metabolic burden is strongly linked to adverse outcomes.

Compared with standard base excess, serial ABE offered clearer prognostic separation. Consistent with this observation, ROC curve analysis at ICU admission demonstrated numerically higher discrimination for ABE compared with BE (AUC 0.671 vs. 0.639), although the difference did not reach statistical significance (ΔAUC = 0.032; *p* = 0.057). This finding suggests that while baseline ABE may provide modest incremental prognostic value over BE, the principal clinical utility of ABE arises from its serial trajectory rather than a single time-point measurement. Survivors typically transitioned to neutral or positive ABE values by 48 h, whereas non-survivors remained persistently negative. This temporal divergence highlights that aggressive early resuscitation may correct hyperlactatemia relatively quickly (2), while recovery from fixed-acid acidosis requires restoration of effective renal perfusion and organ function—processes that often fail in fatal sepsis. Consistent with prior reports, patients with persistently low ABE in our cohort exhibited higher rates of acute kidney injury and renal replacement therapy, reinforcing the close link between unresolved metabolic acidosis and renal dysfunction (11–14).

Renal replacement therapy may influence ABE trajectories through extracorporeal removal of unmeasured anions, potentially leading to numerical improvement in BE and ABE that is not solely attributable to spontaneous metabolic recovery. In our cohort, a minority of patients in the normalizing ABE group (8.6%) required continuous renal replacement therapy (CRRT). Importantly, initiation of CRRT occurred predominantly after the initial 48-h observation window used for ABE trajectory classification, limiting its direct impact on early ABE normalization. Nevertheless, we acknowledge that extracorporeal clearance of fixed acids may contribute to apparent improvement in acid–base parameters independent of lactate clearance, and this distinction should be considered when interpreting ABE dynamics in patients receiving RRT.

Our results extend previous work describing U-shaped associations between acid–base parameters and mortality (1–3). While admission BE and ABE values have been associated with outcomes in prior studies (19–21), our data demonstrate that the direction of ABE change is as important as its absolute value. Patients who corrected an initial ABE deficit had outcomes comparable to those with normal ABE at admission, whereas those who failed to improve experienced substantially higher mortality. This dynamic approach provides clinically actionable information that is not captured by single time-point measurements. Importantly, some patients with initially normal ABE still died, emphasizing that ABE should complement—rather than replace—global clinical assessment.

The observation that patients with normal ABE at admission exhibited higher mortality than those whose ABE normalized over time likely reflects fundamental differences between static and dynamic metabolic states. A normal ABE at presentation does not necessarily indicate preserved metabolic reserve, as early measurements may precede the accumulation of fixed acids or may coexist with severe non-metabolic organ dysfunction. In contrast, normalization of an initially negative ABE represents an active recovery process, reflecting restoration of renal acid handling, improved microcirculatory perfusion, and resolution of fixed-acid burden. Thus, dynamic improvement in ABE appears to capture physiological resilience and adaptive capacity more effectively than a single normal baseline value.

The observed rate of invasive mechanical ventilation (7.2%) among survivors may appear lower than expected for an ICU population with a median SOFA score of approximately 8. However, this finding should be interpreted in the context of the distribution of organ failures and the operational definition of ventilatory support used in this study. In our cohort, admission SOFA scores were frequently driven by circulatory and metabolic components, such as vasopressor-dependent hypotension and acute kidney injury, rather than by primary refractory hypoxemia. Importantly, the reported 7.2% rate reflects only invasive endotracheal intubation and does not include patients managed with non-invasive ventilation or high-flow nasal oxygen, which are commonly employed as first-line respiratory support in contemporary ICU practice. Survivors—who demonstrated earlier metabolic recovery as captured by ABE normalization—appeared less likely to progress to refractory respiratory failure requiring invasive ventilation. Accordingly, the observed ventilation rates reflect favorable disease trajectory and response to early resuscitation rather than lower baseline illness severity.

ABE monitoring may help identify occult hypoperfusion and ongoing metabolic derangements that are not captured by lactate measurements alone. Lactate levels may normalize early during resuscitation due to improved oxygen delivery, reduced adrenergic drive, or enhanced clearance, even while microcirculatory dysfunction and impaired renal acid handling persist. In contrast, a persistently negative ABE reflects the continued presence of fixed acids and unmeasured anions, integrating the cumulative effects of inadequate tissue perfusion, impaired acid excretion, and unresolved metabolic stress. As such, ABE trajectories provide complementary information to lactate by signaling incomplete metabolic recovery despite apparent hemodynamic stabilization.

These findings carry relevant clinical implications. Reliance on lactate clearance alone may lead to premature de-escalation of care in patients who continue to harbor significant fixed-acid acidosis (18). Serial ABE monitoring could serve as a complementary resuscitation target, prompting clinicians to reassess unresolved sources of metabolic derangement such as evolving renal failure, hyperchloremia from chloride-rich fluids, or occult hypoperfusion (22, 23). In patients with persistently negative ABE despite hemodynamic optimization, earlier consideration of metabolic support strategies—including bicarbonate therapy in severe acidosis or timely renal replacement therapy—may be warranted. This rationale is supported by the BICAR-ICU trial, which demonstrated benefit of bicarbonate therapy in patients with severe metabolic acidosis and AKI (24), and by studies linking negative ABE to dialysis requirements in septic shock (14). Although the AKIKI-2 trial did not show an overall mortality benefit with early RRT initiation, stratification by metabolic severity such as ABE may help identify subgroups that could benefit from earlier intervention (25).

This study has several strengths, including a large septic cohort, systematic serial blood gas measurements, and adjustment for key confounders such as SOFA, APACHE II, and baseline eGFR. To our knowledge, this is among the first investigations to characterize sepsis outcomes according to dynamic ABE trajectories rather than static values. Nevertheless, important limitations must be acknowledged. The retrospective single-center design limits causal inference and generalizability. Detailed data on interventions affecting acid–base balance, including bicarbonate administration, fluid composition, and timing of renal replacement therapy, were not uniformly available and may have influenced ABE values. Such factors could lead to partial misclassification of trajectory groups and would be expected to bias results toward the null. In addition, the complex interplay between evolving acute kidney injury and ABE could not be formally explored through mediation analysis. Prospective multicenter studies with granular treatment data are needed to validate these findings and clarify underlying mechanisms.

In summary, persistent negative ABE during early ICU care identifies a metabolically vulnerable sepsis phenotype with substantially increased mortality risk. Monitoring ABE trajectories alongside lactate offers a simple, bedside-accessible approach to refine risk stratification and may inform more individualized metabolic resuscitation strategies.

## Data Availability

The raw data supporting the conclusions of this article will be made available by the authors, without undue reservation.
