# Supplementary material for: Prognostic value of serial alactic base excess measurements in patients with sepsis: a retrospective cohort study
Source: Front Med (Lausanne). 2026 Mar 13;13:1755874. doi: 10.3389/fmed.2026.1755874 (PMC13021659; doi:10.3389/fmed.2026.1755874)
Supplement: Supplementary file 1 [file Table_1.docx]

Suppl. Table 1: Comparisons of Laboratory findings according to the ICU mortality

| **Parameters** | **All patients (n=521)** | **Discharged (n=360)** | **Died (n=161)** | **p-value** |
| --- | --- | --- | --- | --- |
| **Laboratory findings** |  |  |  |  |
| eGFR (mL/min/1.73m²) | 67.9 [45.0–89.0] | 68.8 [48.0–92.0] | 64.7 [41.0–83.0] | **0.010** |
| pH | 7.29 [7.23–7.35] | 7.31 [7.25–7.36] | 7.24 [7.18–7.30] | **<0.001** |
| Bicarbonate (mmol/L) | 22.0 [18.5–25.1] | 22.0 [18.4–25.2] | 22.0 [18.6–24.9] | 0.944 |
| pO₂ (mmHg) | 91 [76–104] | 91 [77–103] | 90 [74–105] | 0.765 |
| pCO₂ (mmHg) | 34.9 [29.8–39.2] | 34.6 [29.5–38.9] | 35.4 [30.4–40.1] | 0.207 |
| Creatinine (mg/dL) | 1.47 [1.10–1.71] | 1.51 [0.90–1.67] | 1.33 [0.90–1.66] | **0.023** |
| SBP (mmHg) | 102 [88–118] | 102 [89–119] | 101 [87–117] | 0.757 |
| DBP (mmHg) | 58 [48–67] | 57 [47–67] | 58 [49–68] | 0.365 |
| Total Bilirubin (μmol/L) | 1.1 [0.8–1.4] | 1.1 [0.8–1.3] | 1.1 [0.8–1.5] | 0.098 |
| Albumin (g/dL) | 2.8 [2.4–3.2] | 2.8 [2.4–3.2] | 2.8 [2.4–3.3] | 0.442 |
| Platelet (x10³/μL) | 203 [154–252] | 205 [156–254] | 198 [149–247] | 0.291 |
| WBC (x10³/μL) | 12.5 [8.4–16.6] | 12.3 [8.2–16.4] | 13.0 [8.9–17.1] | 0.800 |
| Hemoglobin (g/dL) | 10.5 [9.2–11.8] | 10.5 [9.1–11.9] | 10.6 [9.3–11.9] | 0.500 |
| Sodium (mEq/L) | 139.5 [136.2–142.8] | 139.9 [136.5–143.1] | 139.1 [135.8–142.4] | 0.408 |
| Potassium (mEq/L) | 4.2 [3.8–4.6] | 4.2 [3.8–4.6] | 4.1 [3.7–4.5] | 0.521 |
| Chloride (mEq/L) | 102.6 [99.2–106.0] | 102.6 [99.2–106.0] | 102.7 [99.3–106.1] | 0.910 |
| Calcium (mg/dL) | 9.0 [8.4–9.6] | 9.0 [8.4–9.6] | 8.9 [8.3–9.5] | 0.744 |
| Magnesium (mg/dL) | 2.0 [1.8–2.2] | 2.0 [1.8–2.2] | 2.0 [1.8–2.2] | 0.364 |
| CRP (mg/L) | 106 [62–168] | 95 [55–142] | 130 [81–189] | **<0.001** |

Abbreviations: eGFR, estimated glomerular filtration rate; SBP, systolic blood pressure; DBP, diastolic blood pressure; WBC, white blood cell count; CRP, C-reactive protein; ICU, intensive care unit. Continuous variables are reported as median [interquartile range]. Comparisons between groups were performed using Mann–Whitney U test. Statistically significant p-values (<0.05) are indicated in bold.
