# Supplementary material for: Prognostic value of serial alactic base excess measurements in patients with sepsis: a retrospective cohort study
Source: Front Med (Lausanne). 2026 Mar 13;13:1755874. doi: 10.3389/fmed.2026.1755874 (PMC13021659; doi:10.3389/fmed.2026.1755874)
Supplement: Supplementary file 2 [file Table_2.docx]

Suppl. Table 2: Comparisons of Laboratory findings according to the 28-day mortality

| **Parameters** | **Alive**  **n=334** | **Died**  **n=187** | **p-value** |
| --- | --- | --- | --- |
| Laboratory findings |  |  |  |
| eGFR (ml/dk/1.73m²) | 68.55 (5-120) | 66.1 (5-120) | 0.038 |
| pH | 7.31±0.08 | 7.25±0.08 | <0.001 |
| Bicarbonate (mmol/L) | 22.03±4.95 | 21.88±4.92 | 0.750 |
| pO₂ (mmHg) | 90.97±20.06 | 90.03±20.95 | 0.614 |
| pCO₂ (mmHg) | 34.67±7.06 | 35.2±7.08 | 0.414 |
| Creatinine (mg/dL) | 1.5 (0.4-3.45) | 1.41 (0.4-3.52) | 0.198 |
| SBP, mmHg | 102 (60-150) | 101 (60-151) | 0.860 |
| DBP, mmHg | 57.55±13.91 | 57.41±12.51 | 0.910 |
| Total Bilirubin (μmol/L) | 1.1 (1-12.7) | 1.1 (0.98-15) | 0.379 |
| Albumin (g/dL) | 2.8±0.59 | 2.83±0.6 | 0.520 |
| Platelet (x10³/μL) | 203.92±72.85 | 200.33±72.42 | 0.589 |
| WBC (x10³/μL) | 12.4 (2-26.2) | 12.9 (2-25.8) | 0.936 |
| Hemoglobin (g/dL) | 10.49±2.05 | 10.59±1.77 | 0.558 |
| Sodium, mEq/L | 140 (130-149.9) | 139 (130.2-149.6) | 0.360 |
| Potassium, mEq/L | 4.2 (3-5.5) | 4.1 (3-5.5) | 0.474 |
| Chloride, mEq/L | 102.6 (95-110) | 102.5 (95.1-110) | 0.848 |
| Calcium, mg/dL | 9 (7.5-10.5) | 9 (7.5-10.5) | 0.871 |
| Magnesium, mg/dL | 2 (1.5-2.5) | 2 (1.5-2.5) | 0.536 |
| CRP (mg/L) | 94.55 (3-193.7) | 124.9 (13.4-230.1) | <0.001 |

**Abbreviations:** eGFR = Estimated glomerular filtration rate; SBP = Systolic blood pressure; DBP = Diastolic blood pressure; WBC = White blood cell count; CRP = C-reactive protein.

Comparisons between groups were performed using Chi-square or Fisher’s exact test for categorical variables and Mann–Whitney U or Student’s t-test for continuous variables, as appropriate. Statistically significant p-values (<0.05) are indicated in bold.
