# Supplementary material for: Prognostic value of serial alactic base excess measurements in patients with sepsis: a retrospective cohort study
Source: Front Med (Lausanne). 2026 Mar 13;13:1755874. doi: 10.3389/fmed.2026.1755874 (PMC13021659; doi:10.3389/fmed.2026.1755874)
Supplement: Supplementary file 3 [file Table_3.docx]

Suppl. Table 3: Sensitivity Analyses for Alternative ABE Thresholds

| **Threshold** | **Group Sizes (n, %)** | **28-Day Mortality (%)** | **Adjusted HR (95% CI, p-value)** |
| --- | --- | --- | --- |
| 0 mmol/L (Primary) | Persistent low: 188 (36%), Normalizing: 174 (33%), Normal: 159 (30%) | Persistent low: 52.9%, Normalizing: 19.8%, Normal: 26.6% | Persistent low: 2.47 (1.45-4.22, p=0.001) |
| -1 mmol/L | Persistent low: 165 (32%), Normalizing: 190 (36%), Normal: 166 (32%) | Persistent low: 50.3%, Normalizing: 20.5%, Normal: 26.2% | Persistent low: 2.35 (1.38-4.00, p=0.002) |
| -2 mmol/L | Persistent low: 140 (27%), Normalizing: 210 (40%), Normal: 171 (33%) | Persistent low: 48.6%, Normalizing: 21.0%, Normal: 25.7% | Persistent low: 2.12 (1.28-3.51, p=0.003) |

Sensitivity analyses comparing the primary threshold (0 mmol/L) to stricter alternatives (-1 mmol/L and -2 mmol/L) for defining "low" ABE. Group sizes and mortality rates were recalculated after reclassification using alternative ABE thresholds. Adjusted hazard ratios (HR) for persistent low ABE (vs. normalizing) from multivariable Cox models (adjusted for age, sex, eGFR, SOFA, APACHE II). Results demonstrate robustness, with persistent low ABE remaining a significant predictor across thresholds.
